# Risks and benefits associated with the primary functions of artificial intelligence powered autoinjectors

**DOI:** 10.3389/fmedt.2024.1331058

**Published:** 2024-04-05

**Authors:** Marlon Luca Machal

**Affiliations:** Faculty of Medicine and Health Technology, Tampere University, Tampere, Finland

**Keywords:** artificial intelligence, primary functions, autoinjectors, risks, cybersecurity

## Abstract

**Objectives:**

This research aims to present and assess the Primary Functions of autoinjectors introduced in ISO 11608-1:2022. Investigate the risks in current autoinjector technology, identify and assess risks and benefits associated with Artificial Intelligence (AI) powered autoinjectors, and propose a framework for mitigating these risks. ISO 11608-1:2022 is a standard that specifies requirements and test methods for needle-based injection systems intended to deliver drugs, focusing on design and function to ensure patient safety and product effectiveness. ‘KZH’ is an FDA product code used to classify autoinjectors, for regulatory purposes, ensuring they meet defined safety and efficacy standards before being marketed.

**Method:**

A comprehensive analysis of autoinjectors problems is conducted using data from the United States Food and Drug Administration (FDA) database. This database records medical device reporting events, including those related to autoinjectors, reported by various sources. The analysis focuses on events associated with the product code KZH, covering data from January 1, 2008, to September 30, 2023. This research employs statistical frequency analysis and incorporates pertinent the FDA, United Kingdom, European Commission regulations, and ISO standards.

**Results:**

500 medical device reporting events are assessed for autoinjectors under the KZH code. Ultimately, 188 of these events are confirmed to be associated with autoinjectors, all 500 medical devices were seen to lack AI capabilities. An analysis of these events for traditional mechanical autoinjectors revealed a predominant occurrence of malfunctions (72%) and injuries (26%) among event types. Device problems, such as breakage, defects, jams, and others, accounted for 45% of incidents, while 10% are attributed to patient problems, particularly missed and underdoses.

**Conclusion:**

Traditional autoinjectors are designed to assist patients in medication administration, underscoring the need for quality control, reliability, and design enhancements. AI autoinjectors, sharing this goal, bring additional cybersecurity and software risks, requiring a comprehensive risk management framework that includes standards, tools, training, and ongoing monitoring. The integration of AI promises to improve functionality, enable real-time monitoring, and facilitate remote clinical trials, timely interventions, and tailored medical treatments.

## 1 Introduction

Autoinjectors have gained widespread popularity for the treatment of diverse conditions including diabetes, rheumatoid arthritis, multiple sclerosis, and allergies (1). Artificial intelligence (AI) has a transformative impact across various industries, including healthcare, and its integration into autoinjectors has shown significant promise (1–7). AI-powered autoinjectors are expected to offer notable advantages such as improved accuracy in delivering drug and the ability to personalize treatments based on individual patient needs (8). However, these advantages also introduce new risks or can potentially impact existing known risks related to autoinjectors’ problems of administering the drug, necessitating careful consideration.

The updated ISO 11608-1:2022 (9) standard introduced the concept of Primary Functions, which are defined as essential functions that, if they fail to operate correctly, can directly lead to either failure to deliver the drug accurately or cause harm to the patients (9). These Primary Functions are critical for the safe and effective proper function of AI-powered autoinjectors (10–12), and any failure of these functions can have serious consequences for patients. In accordance with ISO 11608-1:2022 (9), the Primary Functions of autoinjectors include facilitating suitable autoinjector Holding force, Cap Removal Force, ensuring a suitable Activation Force, providing an appropriate Extended Needle Length, achieving an optimal Injection Time, ensuring precise Dose Accuracy, and incorporating Needle Guard Lockout for enhanced safety.

This research aims to thoroughly examine the potential risks and benefits associated with AI-powered autoinjectors and provide guidance to manufacturers of autoinjectors and regulatory bodies to address the newly introduced Primary Functions. By identifying and understanding the risks associated with AI powered autoinjectors, it is possible to develop framework for their mitigation. The aim is to provide a framework that can help to minimize the impact of these risks on the Primary Functions of AI-powered autoinjectors, ensuring their proper functioning while maximizing patients’ safety.

To gain insight into risks associated with autoinjectors, valuable information can be extracted from the United States Food and Drug Administration (FDA) Manufacturer and User Facility Device Experience (MAUDE) database (13), which contains records of various device and patient problems related to autoinjectors reported by manufacturers, patients, consumers, and healthcare professionals. The analysis of these reported device and patient problems provides insights into the realm of AI-driven autoinjectors. These insights can be used to improve the safety, usability, and overall reliability of autoinjectors, particularly those powered by AI technology.

By addressing the potential risks associated with AI-powered autoinjectors and implementing effective risk management tools for risk mitigation, this research paves the way for the adoption of AI-powered autoinjectors. This can lead to improved patient outcomes, increased accessibility to personalized treatment, and enhanced overall health care delivery.

## 2 Method and materials

**Figure 1:**
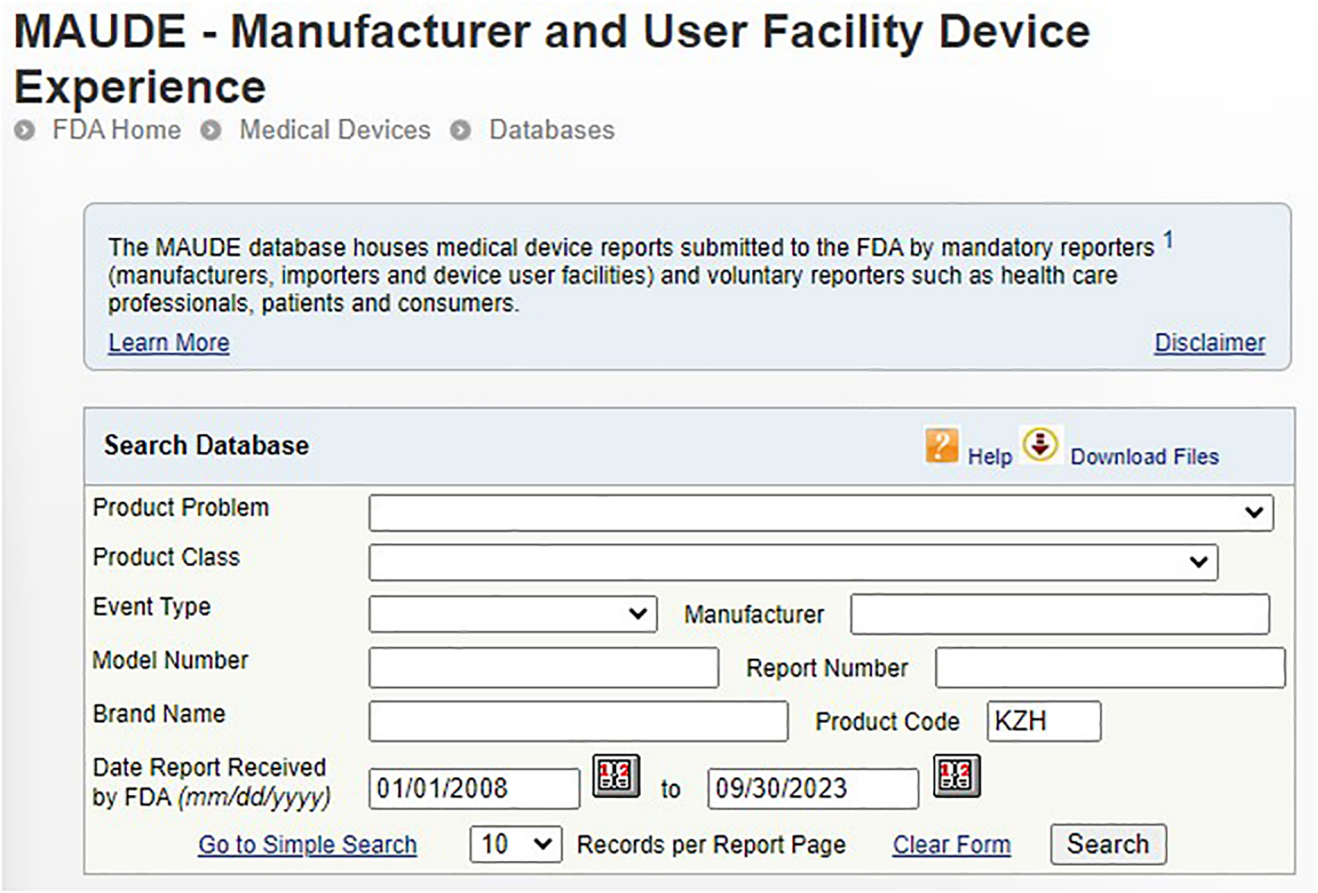
Medical device reporting events search using product code KZH (15) to cover the period of January 1, 2008–September 30, 2023.

To assess the prevailing device and patient problems of autoinjectors, a comprehensive analysis was performed using the data acquired from the MAUDE database of the FDA (13). This database contains records of medical device reporting events related to device and patient problems associated with autoinjectors reported by manufacturers, patients, consumers, and healthcare professionals. The FDA specifically categorizes autoinjectors under the product code KZH (14). Consequently, the assessment focused on the device and patient problems of medical devices associated with the product code KZH (Figure 1). By leveraging this product code, all pertinent device and patient problems of autoinjector were extracted from the medical device reporting events records reported in the MAUDE database. The collection of data spanned a substantial period from January 1, 2008, to September 30, 2023 (Figure 1).

The collected medical device reporting events data contains these datasets: web address, report number, event date, event type, manufacturer, date received, product code, brand name, device problem, patient problem, PMA/PMN number, exemption number, number of events and event text. The data analysis is focused on reported event related to event type, device problem, patient problem, brand name and event text. The choice of this database stems from the FDA's requirement for mandatory reporting by medical device manufacturers. They are obliged to report any adverse events or problems associated with their medical devices, irrespective of whether these adverse events or problems occur within the US or outside of the US. This requirement extends to devices available for sale in the US or those that have obtained clearance for sale in the US. The FDA's regulatory requirements for this reporting is set out in the Medical Device Reporting Regulation, as outlined in the FDA 21 CFR Part 803 (16).

In this research statistical frequency analysis is employed, excel 365 version 2309 that is compatible with Microsoft Excel 97-2003 Worksheet (.xls) was used to generate distribution in percentage. The selection of the frequency analysis helps to understand the distribution of data and identify patterns or anomalies among reported event related to event type, device problem, and patient problem. Distribution analysis is used to examine the frequency of event types, device problem, and patient problem expressed as percentages. The material utilized in this paper comprised relevant regulations and guidance from the FDA, FDA, United Kingdom, European Commission as well as ISO standards.

## 3 Results

**Figure 2:**
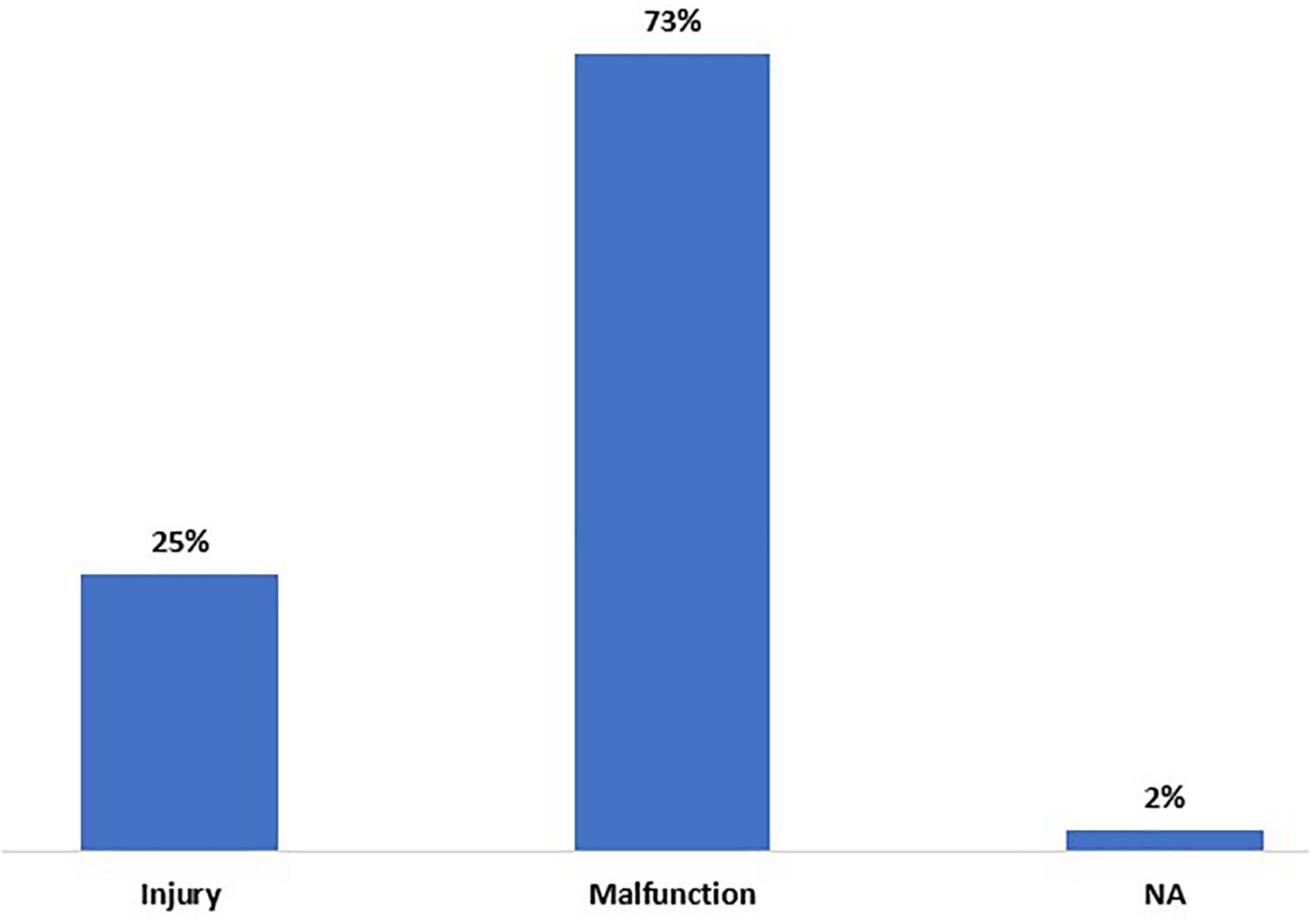
Reported event type of autoinjectors with product code KZH in MAUDE from January 1, 2008, to September 30, 2023.

**Figure 3:**
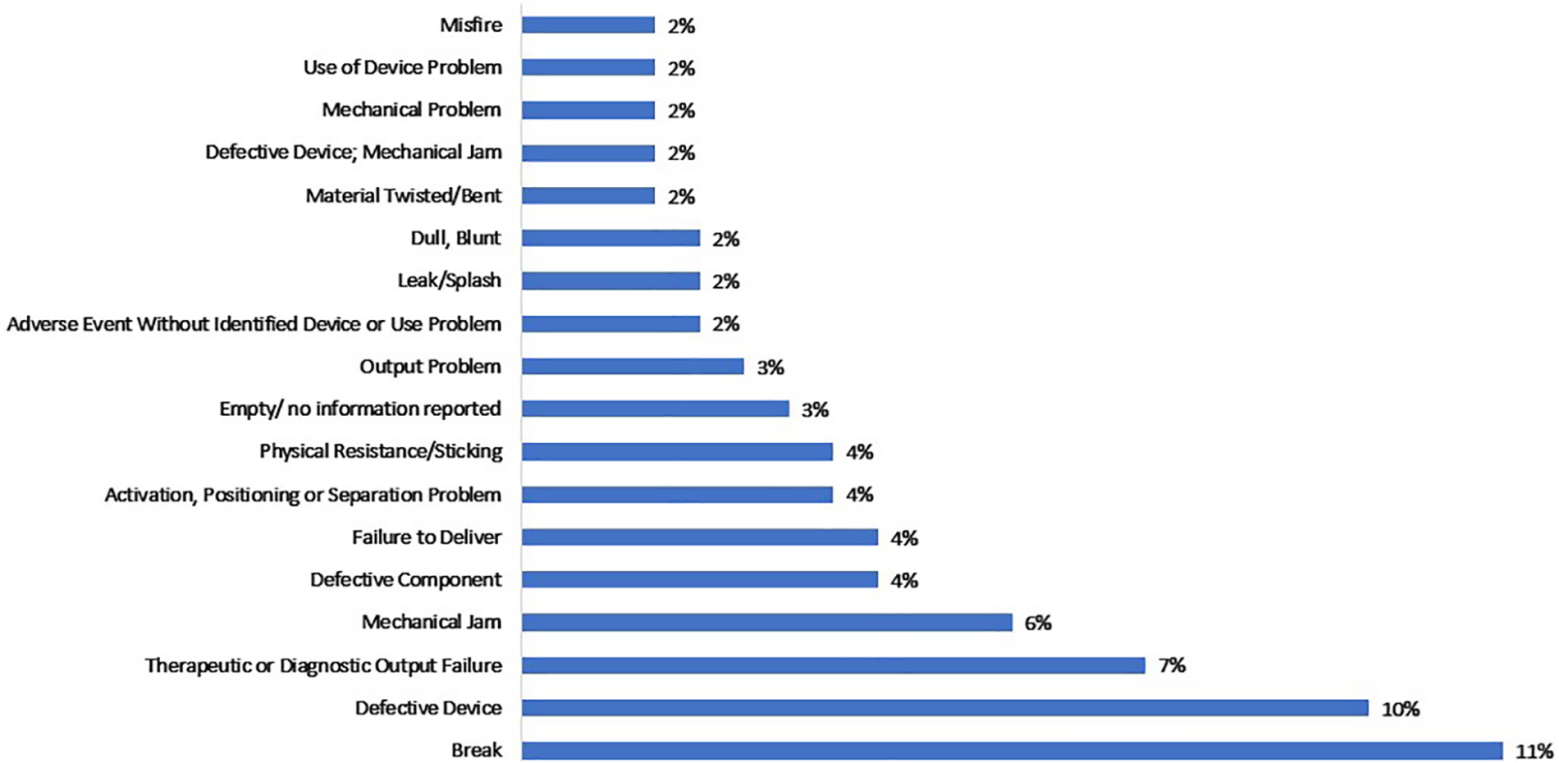
Reported device problems of autoinjectors with product code KZH in MAUDE from January 1, 2008, to September 30, 2023.

**Figure 4:**
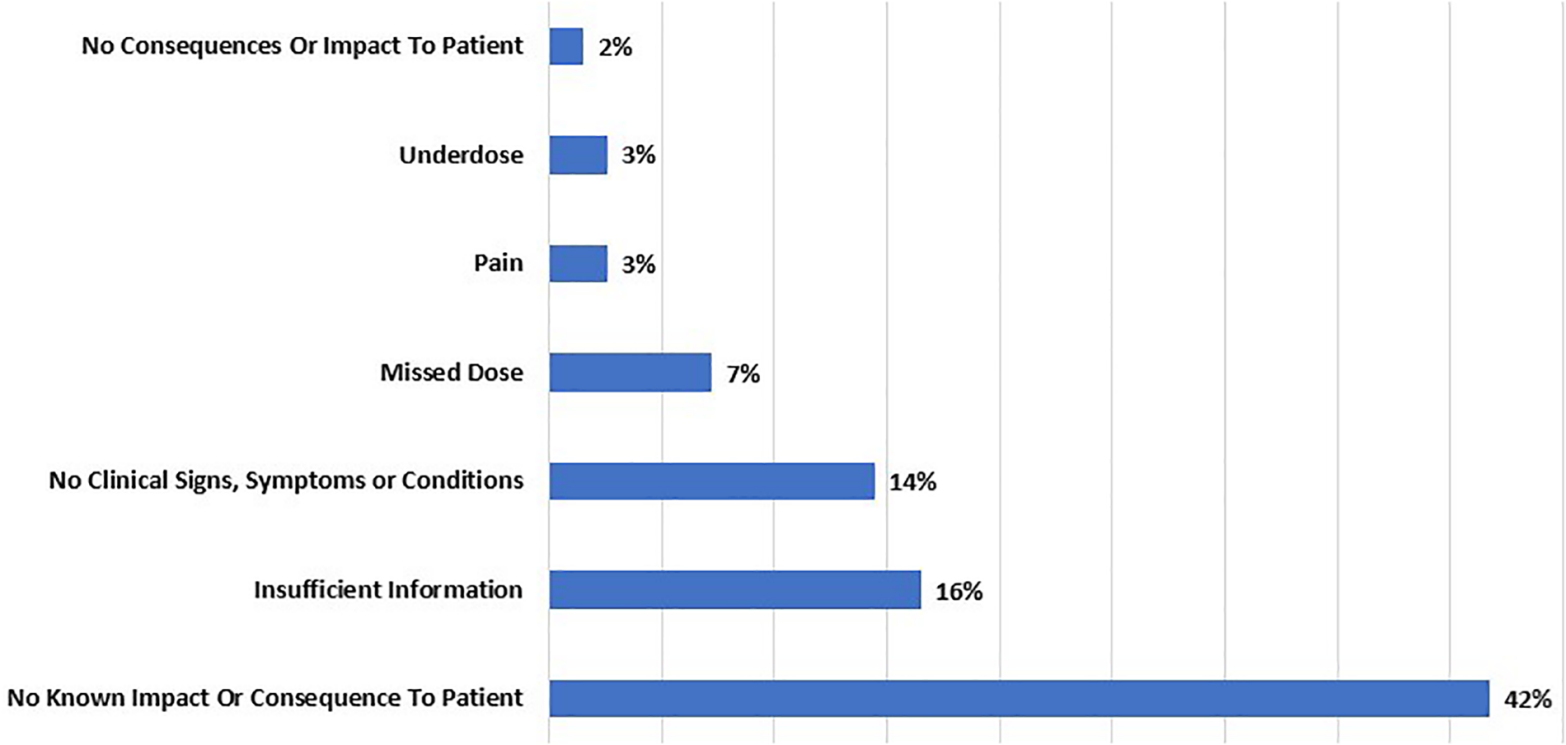
Reported patient problems of autoinjectors with product code KZH in MAUDE from January 1, 2008, to September 30, 2023.

A total of 500 medical device reporting events are reported for autoinjectors using KZH code. Upon reviewing medical device reporting events associated with brand names, it becomes evident to exclude 276 out of 500. This exclusion is due to their association with ACCU-CHEK® LINKASSIST events, which are unrelated to autoinjectors. Out of 224 (500 - 276 = 224) remaining medical device reporting events that are examined, 30 additional events are excluded because they are unrelated to autoinjectors. As a result, the remaining count of medical device reporting events associated with autoinjectors is 194 (224 - 30 = 194). Ultimately, 194 events are confirmed to be related to the autoinjectors based on device brand names and text events. None of these remaining 188 autoinjector have the AI capabilities. The results of the event type, device problem and patient problem are presented in Figures 2–4.

## 4 Discussion

### 4.1 Risk associated with autoinjectors and AI powered autoinjectors

The findings indicated that none of the autoinjectors examined in this study possess AI capabilities. AI-powered autoinjectors can be used in similar fashion like existing traditional mechanical autoinjectors to administer appropriate dose and need to adhere to Primary Functions as per ISO 11608-1:2022 (6). The results in Figures 2–4 provide valuable insights into the safety and effectiveness of autoinjectors. These results encompass data above 2% The results of the event types (Figure 2) show the prevalence of injury of 25% among reported events, which highlights the importance of monitoring and addressing injuries experienced by patients. These injuries may include local skin reactions, pain at the injection site, or other discomforts that can impact patient overall treatment outcomes (17, 18). Furthermore, the substantial prevalence of malfunctions, accounting for 73% of events, accentuates the need for stringent quality control measures and robust device reliability testing throughout the manufacturing process.

The results of the device problems (Figure 3) reveal several critical issues that need attention. Break of 11%, defective device of 10%, mechanical jams of 6%, defective components of 4%, physical resistance/sticking of 4%, leak/splash of 2%, dull, blunt of 2%, defective device, mechanical jam of 2% and mechanical problem 2% count to a total of (11 + 10 + 6+ 4 + 4 + 2+ 2 + 2 + 2 + 2) % = 45%. The total of 45% are related to mechanical issues that pose a substantial risk to patient safety that can result in patients being not able to administer the required prescribed drug, emphasizing the need for improved device design and materials. Therapeutic or diagnostic output failure of 7%, failure to deliver of 4%, output problem of 3%, user device problem of 2% and misfire of 2% that counts to (7 + 4+ 3 + 2+ 2) % = 18% can directly affect the delivery of drug, potentially compromising treatment effectiveness. Empty or no information reported with 3% and adverse event without identified device or use problem count for 2% do not provide any insight into the reported device problems. This underscores the importance of enhancing reporting and data collection to achieve a more comprehensive understanding of problems related to the device.

Patient problems results (Figure 4) show significant proportion of events that counts to 42% had no known impact on patients. However, it's essential to remember that even minor issues that have no impact on patient when they accumulated over time can lead to reduced treatment efficacy, discomfort, and patients’ adherence to administer the drug. Insufficient information counts to 16% which highlights the need for improved reporting and data collection to gain a more comprehensive understanding of patient problems. Additionally, the occurrence of missed of 7%, and underdose of 3% that count to (7 + 3) % = 10%. A 10% of miss and underdose raises concerns related to the delivered dose accuracy which is a Primary Function that affects the overall effectiveness of patients’ treatment outcomes. This issue stems from the inability of the autoinjectors to provide the correct dosage, thereby compromising the efficacy of the drugs.

The pain results, which account for only 3% related to extended needle length which is a Primary Function, may appear relatively low, and it is anticipated that patients may experience some degree of discomfort from needle penetration into the skin for drug delivery. However, if the pain reaches an unbearable level, it becomes necessary for the manufacturer of the autoinjectors to investigate the underlying reasons for such heightened discomfort. Addressing patient concerns should involve improvements in device design and enhanced patient training on the use of the autoinjector. No consequences or impact to patient count to 2% and no clinical signs, symptoms or condition count to 14% which can mean that the use of the autoinjectors didn't result in any harm or negative effects on the patients.

Overall, the analysis of MAUDE medical device reporting events related to traditional mechanical autoinjectors highlights a significant occurrence of malfunctions comprising 73% of all events (Figure 2). Additionally, incidents such as break, defective device, mechanical jams, defective components, physical resistance/sticking, leak/splash and dull, blunt, defective device, mechanical jam and mechanical problem that count to 45%. (Figure 3), while 9.58% of cases involve missed and underdoses (Figure 3). These findings underscore the critical necessity for autoinjector manufacturers to prioritize the enhancement of their autoinjectors designs. It is strongly recommended that they focus on aligning their designs with the FDA's design controls requirements specified in 21 CFR 820.30 (19) and adhere to the guidance provided by ISO 13485 (20), with particular attention to clause 7.3 concerning design and development.

AI failures can significantly impact the design and functionality of autoinjectors, both in their physical and software aspects. The interplay between AI failures and autoinjector design features is crucial for ensuring patient safety and the effectiveness of these devices. Understanding this relationship is vital for developing AI System (AI and autoinjector) that can withstand failures and for creating autoinjectors that prioritize patient well-being. By identifying potential failure points and integrating robust design features, engineers can mitigate risks and enhance the reliability of both AI technology and autoinjector devices. AI-powered autoinjectors, like any medical devices incorporating AI technology, encompass cybersecurity and software risks in addition to the conventional risks of malfunction, injury, device problems, and patient problems identified in the traditional mechanical autoinjectors. These additional cybersecurity and software risks necessitate implementation of risk management process as per ISO 14971 (21), ISO/TR 24971 (22), TIR105 (23) and IEC 62304 (24) to ensure patient safety by mitigating the potential risks associated with cybersecurity breaches and potential software failures. Ensuring the safety and intended use of AI- powered autoinjectors relies on effective management of the risks associated with the Primary Functions underlined in ISO 11608-1:2022 (9).

### 4.2 Mitigating risks associated with AI-powered autoinjectors

ISO 14971 (21), ISO/TR 24971 (22), TIR105 (23) and IEC 62304 (24), IEC 80001-1 (25), NIST SP 800-53 (26), NISTIR 8259A (27), UL 2900-1 (28), AAMI TIR57 (29), AAMI TIR97 (30) standards, and FDA Post-Market Management of Cybersecurity in Medical Devices (31) are important in the context of addressing cybersecurity and software risks in medical devices that use software and wireless connections. Moreover, specific regulatory requirements may be applied in different countries (32–37), which should also be considered during the development and use of AI-powered autoinjectors. Table 1 provides a framework for the Primary Functions that require rigorous risk management in the context of autoinjectors. ISO 14971 clause 7 risk control (21), AAMI TIR105 clause 6 risk control (23) and IEC 62304 clause 7 Software risk management process (24) emphasizes the importance of risk control through inherent safety by design, manufacturing, and user information. For AI-powered autoinjectors, risk management incorporates cybersecurity considerations and adheres to recognized standards (21–31).

**Table 1:** Risk management framework for autoinjector's primary functions.

Primary Functions	Cybersecurity breaches & potential software failures	Design	Manufacturing	Informing users	Postproduction
** **	ISO 14971 clause 7 risk control (21)				
	AAMI TIR105 clause 6 risk control (23)				
Holding Force	(21–29)	DFMEA, FTA	PFMEA	Training & IFU	PMS (31–33, 38, 39)
Cap Removal Force	(21–29)	DFMEA, FTA	PFMEA	Training & IFU	PMS (31–33, 38, 39)
Activation Force	(21–29)	DFMEA, FTA	PFMEA	Training & IFU	PMS (31–33, 38, 39)
Extended Needle Length	(21–29)	DFMEA, FTA	PFMEA	Training & IFU	PMS (31–33, 38, 39)
Injection Time	(21–29)	DFMEA, FTA	PFMEA	Training & IFU	PMS (31–33, 38, 39)
Dose Accuracy	(21–29)	DFMEA, FTA	PFMEA	Training & IFU	PMS (31–33, 38, 39)
Needle Guard Lockout	(21–29)	DFMEA, FTA	PFMEA	Training & IFU	PMS (31–33, 38, 39)

Ensuring the success of the Primary Functions of an AI-powered autoinjector involves a thorough assessment of cybersecurity vulnerabilities and potential software failures at multiple stages of its lifecycle (21–29), including design (19, 20, 30), manufacturing (19), informing user (40–43), and postproduction processes (31–33, 38, 39). The success of the Primary Functions of an autoinjector can be ensured by establishing a comprehensive risk management framework (Table 1) for the autoinjector's entire lifecycle. It is vital to use the conventional risk management tools such as Failure Mode and Effects Analysis (FMEA) and Fault Tree Analysis (FTA) to ensure the robustness of the design, and Process Failure Mode and Effects Analysis (PFMEA) to overcome the manufacturing challenges (44–47). The conventional autoinjector risk management tools like FMEA, FTA, and PFMEA can serve as valuable mechanism for addressing both the identified device problems (Figure 3) and potential problems that may arise as a result of cybersecurity breaches, software failure, poor design, manufacturing challenges, insufficient user training, and inadequate IFU for AI-powered autoinjectors. The device problems and patient problems can become apparent during the postproduction phase when the AI-powered autoinjectors are used in large scale by patients. These problems are collected through known PMS (31–33, 38, 39) activities and used to assess risk control measure effectiveness, identify new risks and improve the AI-powered autoinjectors.

### 4.3 Benefits of the AI-powered autoinjectors

The integration of artificial intelligence into autoinjectors has the capacity to improve the Primary Functions of these devices by effectively tackling PMS issues stemming from event types, device problems and patient problems. This is especially pertinent to issues related to insufficient information, missed dose and underdose events. This AI integration can be crucial in safeguarding patients’ lives, particularly when using autoinjector with medications that necessitate immediate and significant impacts on the patient if the dosage is not administered correctly. The risk of dose inaccuracy, whether due to underdosing or overdosing, can accumulate over years of using non-AI-powered autoinjectors, posing a significant health hazard. Such inaccuracies in dosage can result in patients not receiving the full prescribed dose, ultimately affecting the efficacy of the treatment. AI-powered autoinjectors present effective solutions by offering real-time monitoring and enables timely interventions, detect irregularities, reducing pain by enhancing injection-site using personalized approaches and adjust the injections to prevent harm (8, 17, 18). With the FDA's increasing support for remote clinical trials, enabling participants to engage from their homes through telemedicine, digital health tools, and remote monitoring (48, 49), there is an opportunity to incorporate AI-powered autoinjectors into these trials. This integration has the potential to significantly reduce the need for unnecessary hospital or clinical site visits. The AI capabilities of autoinjectors enable accurate data tracking, ensure prompt corrective measures, and increase patient safety (50–52). Furthermore, they can provide instructions to patients through an application on smart phones or tablets, empowering them to manage their treatments more effectively, revolutionizing patient care, and elevating their healthcare experience (53–55).

## 5 Recommendations

Given the challenges associated with AI-powered autoinjectors, the following recommendations can be proposed.

1. Adaptation of existing regulations: Modify current regulations governing medical devices to incorporate specific provisions for AI-powered autoinjectors. This may involve updating definitions, classification criteria, and testing requirements to address the unique characteristics and functionalities of AI-driven devices. One approach to strengthen existing regulations is by advocating for the update of ISO 11608-1 or the introduction of a new series specifically tailored to AI-powered autoinjectors.
2. Guidelines for AI algorithm validation: Develop comprehensive guidelines for validating the AI algorithms embedded in autoinjectors. These guidelines should outline rigorous testing protocols to assess the safety, efficacy, and reliability of AI algorithms in real-world scenarios. Incorporating principles of transparency, interpretability, and reproducibility will be essential.
3. Data governance and privacy standards: Establish robust data governance and privacy standards to govern the collection, storage, and utilization of patient data by AI-powered autoinjectors. Ensure compliance with existing data protection regulations and promote transparency regarding data usage to build patient trust and confidence.
4. Interoperability and compatibility requirements: Define interoperability and compatibility requirements to facilitate seamless integration of AI-powered autoinjectors with existing healthcare infrastructure and electronic medical records systems. Standardized data formats and communication protocols should be mandated to enable secure data exchange and interoperability across different platforms.
5. Continuous monitoring and surveillance: Implement mechanisms for continuous monitoring and surveillance of AI-powered autoinjectors post-market to detect and address any potential safety issues or performance degradation over time. Utilize real-world data analytics and feedback mechanisms to ensure timely intervention and risk mitigation.
6. Human factors and user interface design: Emphasize the importance of human factors and user interface design in the regulatory evaluation of AI-powered autoinjectors. Ensure that devices are intuitive, user-friendly, and accessible to individuals with diverse abilities to enhance usability and reduce the risk of user errors.
7. Collaboration and stakeholder engagement: Foster collaboration and engagement among regulators, industry stakeholders, healthcare providers, and patient advocacy groups to facilitate knowledge sharing, consensus building, and alignment of regulatory priorities. Establish platforms for ongoing dialogue and exchange of best practices to promote innovation while safeguarding patient safety.
8. Agile regulatory frameworks: Develop agile regulatory frameworks capable of adapting to rapid technological advancements in AI. Implement mechanisms for expedited review and approval of AI-powered autoinjectors that demonstrate substantial clinical benefits while maintaining rigorous safety standards.

## 6 Conclusion

Traditional mechanical autoinjectors and AI-powered autoinjectors share same newly introduced Primary Functions in ISO 11608-1:2022. The reason is that their common objective is to enable patients in administering prescribed drug in alignment with these defined Primary Functions. The analysis of event types, device problems, and patient problems associated with traditional mechanical autoinjectors (Figures 2–4) underscores the necessity for strict quality control and robust device reliability testing during manufacturing. It highlights the importance of enhancing device design and materials, as well as addressing the issue of insufficient information by improving reporting and data collection processes. The identified malfunctions, injuries, device and patient problems associated with traditional mechanical autoinjectors can serve as insight when developing AI-powered autoinjectors.

AI-driven autoinjectors, similar to all medical devices incorporating AI technology, encompass cybersecurity and software-related risks in addition to the conventional risks such as malfunctions, injuries, device issues, and patient problems identified in traditional mechanical autoinjectors. Ensuring the success of the Primary Functions of an AI-powered autoinjector involves a thorough assessment of cybersecurity breaches and potential software failures at multiple stages of its lifecycle, including design, manufacturing, user information management, and postproduction processes.

The Risk management framework for autoinjector's primary functions is introduced aiming to offer guidance to mitigate risks of AI powered autoinjectors in selecting suitable standards to address cybersecurity risks, design, manufacturing concerns and issues that can be captured during PMS. It underscores the importance of using risk management tools, adequately informing users through training and IFU. Furthermore, the framework includes a postproduction phase to gain insights into new risks and assess the effectiveness of risk control measures when AI-powered autoinjectors are widely used by patients. These insights are gathered through established PMS activities, contributing to the enhancement of AI-powered autoinjectors.

The integration of AI into autoinjectors holds great potential for enhancing their Primary Functions, particularly addressing issues identified through PMS, such as malfunctions, injuries, device and patient problems. This is especially crucial when dealing with drugs requiring immediate or precise dose administration, as inaccuracies in dosage, whether underdosing or overdosing, can accumulate over time and pose significant health risks. AI-powered autoinjectors provide the capability for real-time monitoring, making them suitable for use in remote clinical trials. They can facilitate timely interventions, reduce pain, and offer personalized approaches, thereby mitigating harm and ensuring patient safety. As AI continues to advance, the imperative to protect patient safety and enhance treatment outcomes remains of utmost importance. AI-powered autoinjectors represent a leap forward in achieving these goals, promising a future where healthcare is more effective, efficient, and patient-centered.

Efforts to strengthen regulations for AI-powered autoinjectors include updating existing regulations like ISO 11608-1 or introducing new series tailored to these devices. Additionally, comprehensive guidelines for AI algorithm validation, robust data governance, interoperability standards, continuous monitoring, user-friendly design, stakeholder collaboration, and agile regulatory frameworks are essential for ensuring safety and efficacy in their usage.

## Data Availability

The datasets presented in this study can be found in online repositories. The names of the repository/repositories and accession number(s) can be found in the article/Supplementary Material.
